# Post-transcriptional regulation of meiotic genes by a nuclear RNA silencing complex

**DOI:** 10.1261/rna.044479.114

**Published:** 2014-06

**Authors:** Emily D. Egan, Craig R. Braun, Steven P. Gygi, Danesh Moazed

**Affiliations:** 1 Howard Hughes Medical Institute, Harvard Medical School, Boston, Massachusetts 02115, USA; 2 Department of Cell Biology, Harvard Medical School, Boston, Massachusetts 02115, USA

**Keywords:** Red1, mRNA degradation, H3K9 methylation, meiosis, nuclear bodies, fission yeast

## Abstract

RNA is a central component of gene-silencing pathways that regulate diverse cellular processes. In the fission yeast *Schizosaccharomyces pombe*, an RNA-based mechanism represses meiotic gene expression during vegetative growth. This pathway depends on the zinc finger protein Red1, which is required to degrade meiotic mRNAs as well as to target histone H3 lysine 9 (H3K9) methylation, a repressive chromatin mark, to a subset of meiotic genes. However, the mechanism of Red1 function is unknown. Here we use affinity purification and mass spectrometry to identify a Red1-containing nuclear RNA silencing (NURS) complex. In addition to Red1, this complex includes the Mtl1, Red5, Ars2, Rmn1, and Iss10 proteins and associates with several other complexes that are involved in either signaling or mediating RNA silencing. By analyzing the effects of gene knockouts and inducible knockdown alleles, we show that NURS subunits regulate RNA degradation and H3K9 methylation at meiotic genes. We also identify roles for individual NURS subunits in interactions with Mmi1, an RNA-binding protein that marks meiotic RNAs for destruction, and the nuclear exosome RNA degradation complex. Finally, we show that the levels of H3K9 methylation at meiotic genes are not sufficient to restrict RNA polymerase II access or repress gene expression during vegetative growth. Our results demonstrate that Red1 partners with other proteins to silence meiotic gene expression at the post-transcriptional level. Conservation of a NURS-like complex in human cells suggests that this pathway plays an ancient and fundamental role in RNA silencing.

## Abstract (precis)

The authors define a multiprotein nuclear RNA silencing (NURS) complex that mediates silencing of meiotic genes during vegetative growth in the fission yeast *S. pombe*. Meiotic gene silencing occurs post-transcriptionally through recruitment of the exosome complex to promote RNA degradation. Extensive interaction analysis and functional characterizations link the NURS complex to specific RNA-binding and processing proteins and also chromatin modification machinery.

## INTRODUCTION

RNA-dependent mechanisms play roles in gene silencing both at the transcriptional (TGS) and post-transcriptional (PTGS) levels in a broad range of organisms. These mechanisms are often regulated by specific RNA features that determine whether a nascent transcript becomes a functional RNA, is degraded, or feeds back on chromatin to silence transcription. In some cases, the RNA is processed into various classes of small RNAs that are associated with RNA interference (RNAi) pathways. In the fission yeast, *Schizosaccharomyces pombe*, small interfering RNAs (siRNAs) derived from noncoding centromeric transcripts are loaded onto the Argonaute (Ago1) subunit of the RNA-induced transcriptional silencing (RITS) complex. RITS then recruits the Clr4–Rik1–Cul4 (CLRC) methyltransferase complex to catalyze the di- and trimethylation of histone H3 lysine 9 (H3K9) to initiate heterochromatin formation at specific chromosome regions (Moazed 2009). Another mechanism involving the Trf4–Air2–Mtr4 polyadenylation (TRAMP) complex broadly couples the transcription of cryptic unstable transcripts (CUTs) and other classes of aberrant RNA to degradation by the exosome, a multisubunit 3′ to 5′ exonuclease (LaCava et al. 2005; Houseley et al. 2006; Chlebowski et al. 2013). The TRAMP complex is also required for efficient silencing of *S. pombe* centromeric transcripts that are targeted by RNAi (Buhler et al. 2007; Reyes-Turcu et al. 2011). Long noncoding RNAs (lncRNAs), akin to fission yeast centromeric RNAs, have recently emerged as important mediators of gene silencing in plant and animal cells, particularly during development and differentiation (Lee 2012; Wierzbicki 2012).

The onset of meiosis is a major developmental transition in *S. pombe* and involves the induction of hundreds of genes (Mata et al. 2002). Several interconnected mechanisms silence meiotic gene expression during vegetative growth. Many of these genes, including the master regulator *mei4*^+^, are transcribed during vegetative growth, but the mRNAs are rapidly degraded (Harigaya et al. 2006). This RNA elimination pathway depends on Mmi1, a member of the conserved YTH family of RNA-binding proteins. Mmi1 recognizes determinant of selective removal (DSR) sequences and targets mRNAs for exosome-mediated degradation (Harigaya et al. 2006; St-Andre et al. 2010; Yamanaka et al. 2010; Yamashita et al. 2012). RNA elimination also requires components of the 3′-end processing machinery, including the cleavage factors Rna15 and Pcf11, the poly(A) polymerase Pla1, and the poly(A)-binding protein Pab2 (St-Andre et al. 2010; Yamanaka et al. 2010). In addition, the degradation of Mmi1 targets depends on Red1, a zinc finger-containing protein (Sugiyama and Sugioka-Sugiyama 2011). Despite considerable progress in recent years, we still lack a full understanding of how RNA features and protein factors work together to recruit the exosome and promote meiotic RNA degradation.

In addition to their post-transcriptional regulation by the RNA elimination pathway, a subset of meiotic genes, including *mei4*^+^ and *ssm4*^+^, possesses low levels of histone H3K9 methylation during vegetative growth, suggesting that they may also be subject to transcriptional gene silencing (Cam et al. 2005). The establishment of H3K9 methylation at these meiotic genes is largely RNAi-independent, although RITS subunits are detected at *mei4*^+^ and *ssm4*^+^ gene loci, and RNAi mutants may exhibit a slight increase in the levels of these RNAs (Cam et al. 2005; Hiriart et al. 2012; Zofall et al. 2012; Tashiro et al. 2013). It has been proposed that Red1 directly recruits CLRC (Zofall et al. 2012), but this mechanism does not explain how H3K9 methylation is targeted to specific Mmi1/Red1-regulated genes. While both Mmi1 and Red1 are required for histone H3K9 methylation, the Mmi1 binding site, DSR, is sufficient for RNA degradation but not for H3K9 methylation in some genomic contexts (Zofall et al. 2012; Tashiro et al. 2013). Therefore, CLRC recruitment may involve additional protein factors or DNA or RNA sequence elements (Tashiro et al. 2013). Red1 also functions in an RNAi-dependent silencing pathway that establishes heterochromatin at some meiotic genes, transmembrane domain protein-encoding genes, and transposons upon exosome inactivation or exposure to specific growth conditions (Yamanaka et al. 2013). H3K9 methylation at meiotic genes disappears upon entry into meiosis, and it was therefore suggested that these sites of H3K9 methylation are analogous to regions of facultative heterochromatin found in multicellular organisms (Trojer and Reinberg 2007; Zofall et al. 2012; Tashiro et al. 2013).

Here we use affinity purification and mass spectrometry to identify a multiprotein Red1-containing complex. We find that Red1 associates with an Mtr4-like helicase Mtl1 (SPAC17H9.02), two zinc finger proteins, Red5 (SPBC337.12) and Ars2 (SPBC725.08), the RNA recognition motif (RRM)- containing protein Rmn1 (SPBC902.04), and a serine- and proline-rich protein Iss10 (SPAC7D4.14c). These Red1-interacting proteins co-purify each other, indicating that they form a complex, which we name the nuclear RNA silencing (NURS) complex. Consistent with the purification results, all members of the NURS complex colocalize in nuclear foci. However, these nuclear bodies do not colocalize with the *mei4*^+^ locus, suggesting that their formation occurs post-transcriptionally. By generating gene knockouts of *iss10*^+^ and *rmn1*^+^ and inducible knockdown alleles of the essential genes *mtl1*^+^, *red5*^+^, and *ars2*^+^, we show that Mtl1, Iss10, and Red5 are required for meiotic mRNA elimination and H3K9 methylation at meiotic genes. In addition, purification of Red1 after knockdown of Mtl1 or deletion of *iss10*^+^ revealed that Mtl1 is required for exosome recruitment, while Iss10 helps bridge the NURS complex to Mmi1. We also find that the expression of meiotic genes is not affected by the absence of heterochromatic silencing machinery and that the H3K9 methylation at meiotic genes does not restrict RNA polymerase (Pol) II access, suggesting that the presence of this histone mark at meiotic gene loci is not associated with the assembly of functional heterochromatin. Our results suggest that the NURS complex links various RNA processing signals to exosome-mediated nuclear RNA degradation. Very recently, an independent report (Lee et al. 2013) described the purification of a similar Red1-containing complex, in agreement with the findings reported here.

## RESULTS

### Red1 associates with five other proteins to form a nuclear RNA silencing (NURS) complex

**FIGURE 1.**
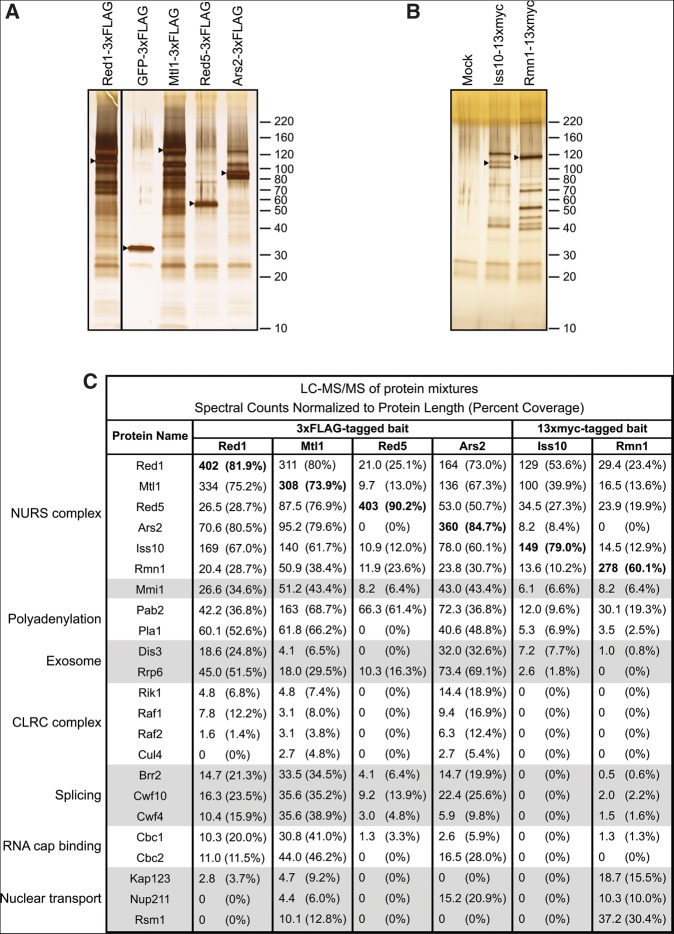
Purification of Red1 and identification of the NURS complex. (*A*,*B*) Silver-stained SDS polyacrylamide gels showing proteins recovered by purification of the indicated 3×FLAG- or 13×myc-tagged protein. Control purifications were performed using extracts from cells expressing a transgene-encoded GFP-3×FLAG protein or no tagged protein (mock). Arrowheads indicate the positions of tagged proteins. Molecular weight markers (kilodalton, kDa) are shown on the *right*. (*C*) Results of liquid chromatography-tandem mass spectrometry (LC-MS/MS) analysis of protein mixtures displayed as normalized spectral counts (raw number of spectral counts divided by protein length in amino acids multiplied by 1000) and percent coverage in parentheses. The values for each tagged protein bait are highlighted by bold type. NURS, nuclear RNA silencing; CLRC, Clr4–Rik1–Cul4.

To gain insight into the dual functions of Red1 in promoting mRNA degradation as well as H3K9 methylation, we purified FLAG-tagged Red1 and identified interacting proteins by mass spectrometry (Fig. 1A,C). We identified five proteins that reproducibly co-purified with Red1. The most abundant of these was SPAC17H9.02/Mtl1, an essential Mtr4-like helicase (Fig. 1C). However, unlike Mtr4, this protein was not previously identified in purifications of the TRAMP complex (Buhler et al. 2007; Keller et al. 2010; Zhang et al. 2011). Two zinc finger-containing proteins were also detected. SPBC337.12/Red5 was recently suggested to possess a YTH domain like Mmi1 and found to be required for meiotic mRNA elimination (Sugiyama et al. 2013). SPBC725.08/Ars2 is orthologous to mammalian and *Drosophila* Ars2 and *Arabidopsis* SERRATE. In these systems, Ars2/SERRATE interacts with the RNA cap-binding complex and regulates siRNA and micro (mi)RNA biogenesis (Yang et al. 2006; Laubinger et al. 2008; Gruber et al. 2009; Sabin et al. 2009; Raczynska et al. 2013). Mammalian ARS2 also functions in an exosome-recruitment pathway involved in the degradation, transcription termination, and 3′-end processing of diverse RNAs (Gruber et al. 2009, 2012; Andersen et al. 2013; Hallais et al. 2013). Another strongly associated protein was SPAC7D4.14c/Iss10, a Ser/Pro-rich protein recently found to function in meiotic mRNA elimination (Yamashita et al. 2013). Finally, we identified SPBC902.04/Rmn1, an RRM-containing protein implicated in mRNA export (Cho et al. 2012).

**FIGURE 2.**
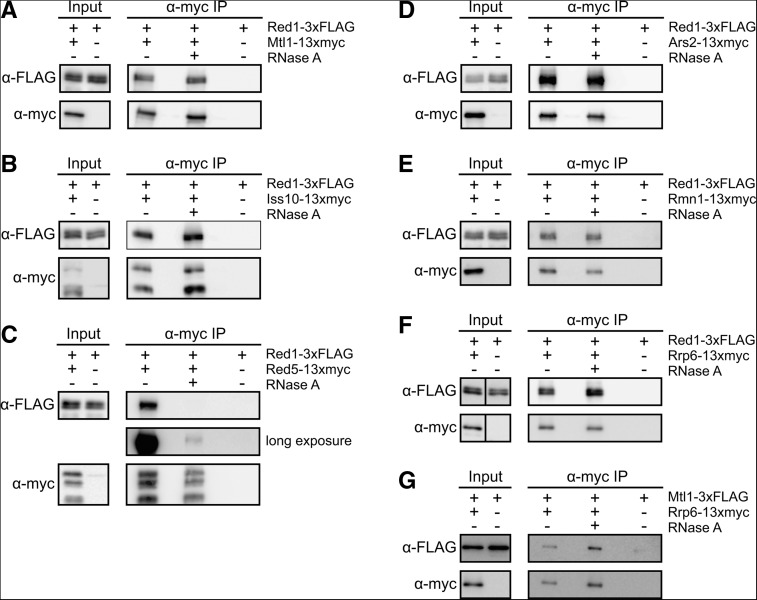
The effect of RNase treatment on the integrity of the NURS complex and its association with the exosome. (*A–E*) Western blots showing co-immunoprecipitation of Red1-3×FLAG by 13×myc-tagged Mtl1, Iss10, Red5, Ars2, or Rmn1 in the presence or absence of RNase A. (*F*,*G*) Western blots showing co-immunoprecipitation of Red1-3×FLAG and Mtl1-3×FLAG by Rrp6-13×myc in the presence or absence of RNase A.

We tagged and purified each Red1-interacting protein and identified co-purifying proteins by mass spectrometry (Fig. 1A–C). We found that Red1 co-purified with each one of its interacting partners (Fig. 1C). Furthermore, all Red1-interacting proteins co-purified with each other, except that Red5-3×FLAG and Rmn1-13×myc purifications did not yield Ars2. However, the fact that Ars2-3×FLAG purifications contained Red5 and Rmn1 suggests that the C-terminal tags on Red5 and Rmn1 may interfere with their interaction with Ars2 (Fig. 1C). We confirmed the interaction of Mtl1, Iss10, Red5, Ars2, and Rmn1 with Red1 by co-immunoprecipitation (Supplemental Fig. S1), and we tested the RNA dependence of these interactions by treating cell extracts with RNase A (Fig. 2A–E). While Red1 binding to Mtl1, Iss10, Ars2, and Rmn1 was not RNase-sensitive (Fig. 2A,B,D,E), the Red1–Red5 interaction was greatly diminished with RNase treatment (Fig. 2C). This finding suggests that Red5 may help recruit NURS by directly binding to RNA targets. Alternatively, NURS association with RNA may trigger a conformational change required for Red5 to make protein–protein interactions.

Red1 and its interacting partners co-purified Mmi1 and components of the exosome and the cleavage and polyadenylation machinery (Fig. 1C; Supplemental Tables S1, S2), as expected based on previous studies (Sugiyama and Sugioka-Sugiyama 2011; Zofall et al. 2012). In good agreement with published data, Red1 co-immunoprecipitated the exosome subunit Rrp6 in an RNA-independent manner, and we found that the same was the case for Mtl1 (Fig. 2F,G; Sugiyama and Sugioka-Sugiyama 2011). Splicing factors were also identified in several of the purifications (Fig. 1C; Supplemental Tables S1, S2). In addition, purifications of Red1, Mtl1, and Ars2 contained significant amounts of the RNA cap-binding complex subunits Cbc1/SPAC6G10.07 and Cbc2/SPBC13A2.01c (Fig. 1C). Finally, consistent with its role in mRNA export (Cho et al. 2012), Rmn1 co-purified several proteins involved in nucleocytoplasmic transport, including the importin Kap123, the nucleoporin Nup211, and the mRNA export factor Rsm1 (Fig. 1C; Supplemental Table S2).

Red1 has been shown to interact with the histone H3 methyltransferase Clr4 by co-immunoprecipitation, and it has been suggested that Red1 binds nascent RNA transcripts and directly recruits CLRC to meiotic gene loci (Zofall et al. 2012; Tashiro et al. 2013). Although we did not detect Clr4 itself in our purifications, we identified multiple other members of the CLRC complex in purifications of Red1, Mtl1, and Ars2 (Fig. 1C).

Together these data suggest that Red1 forms a complex with five other proteins, which we term the nuclear RNA silencing (NURS) complex. Several members of this complex contain nucleic acid-binding domains, which may mediate interactions with nascent RNA or DNA to enable RNA degradation and H3K9 methylation.

### The NURS complex localizes to nuclear foci that do not colocalize with the *mei4*^+^ locus

**FIGURE 3.**
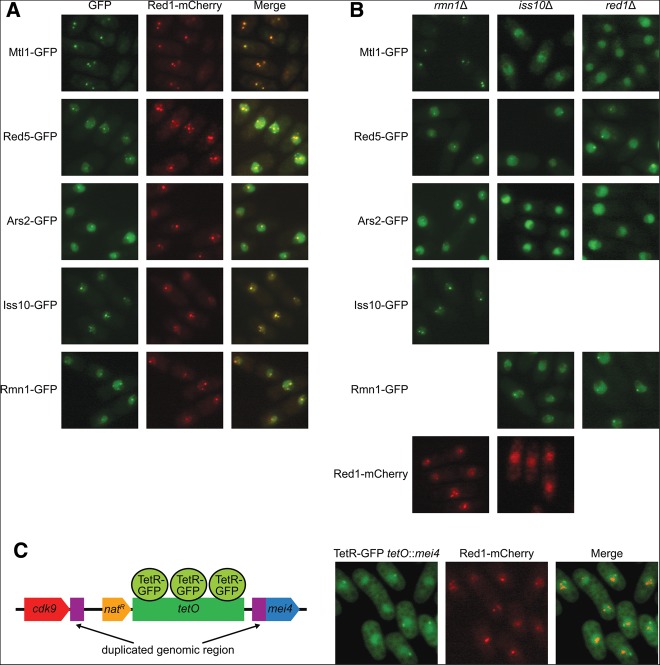
Subcellular localization of subunits of the NURS complex. (*A*) Fluorescence microscopy of live cells expressing the indicated GFP-tagged protein and Red1-mCherry. (*B*) Fluorescence microscopy of live cells expressing the indicated GFP-tagged protein in the absence of *rmn1^+^*, *iss10^+^*, or *red1^+^* or Red1-mCherry in the absence of *rmn1^+^* or *iss10^+^*. (*C*) Diagram of the modified *mei4*^+^ locus (*tetO::mei4*) with integrated nourseothricin (nat) resistance marker and *tetO* repeats bound by GFP-tagged Tet repressor (TetR) and fluorescence microscopy of live cells harboring a *tetO* array at the *mei4*^+^ locus and expressing TetR-GFP and Red1-mCherry.

Red1 was originally identified in a screen for proteins that form nuclear bodies and was shown to localize in one to four nuclear foci in vegetative cells (Sugiyama and Sugioka-Sugiyama 2011). To determine whether other members of the NURS complex share this pattern of localization, we examined live cells expressing each GFP-tagged NURS subunit together with Red1-mCherry by fluorescence microscopy. We found that Mtl1, Red5, Ars2, Iss10, and Rmn1 colocalized with Red1 in nuclear foci (Fig. 3A; Sugiyama et al. 2013; Yamashita et al. 2013). In addition to concentrating in foci, Red5, Ars2, and Rmn1 localized throughout the nucleus (Fig. 3A). Mmi1, the exosome subunits Rrp6 and Dis3, and components of the polyadenylation machinery Pab2, Pla1, and Pcf11 have also been reported to colocalize with Red1 in nuclear bodies (Yamanaka et al. 2010; Sugiyama and Sugioka-Sugiyama 2011). These structures have been suggested to be analogous to mammalian cleavage bodies, which contain cleavage and polyadenylation factors and newly synthesized RNA (Schul et al. 1996; Sugiyama and Sugioka-Sugiyama 2011).

We then investigated whether the localization of the NURS complex was dependent on any of its nonessential components. Deletion of *rmn1*^+^ did not affect the localization of Mtl1-GFP, Red5-GFP, Ars2-GFP, Iss10-GFP, or Red1-mCherry (Fig. 3B) or the colocalization of the other members of the complex with Red1 (data not shown). However, deletion of *iss10*^+^ resulted in the loss of Red1-mCherry, Mtl1-GFP, and Ars2-GFP from nuclear foci and their even distribution throughout the nucleus (Fig. 3B). Deletion of *red1*^+^ also caused Mtl1-GFP and Ars2-GFP to mislocalize (Fig. 3B). However, we observed Iss10 protein accumulation depends on Red1, as was recently reported (Yamashita et al. 2013). Thus, the mislocalization of Mtl1 and Ars2 in *red1*Δ cells may be due to the absence of Iss10. Iss10 protein levels were unaffected by *rmn1*^+^ deletion, and none of the other members of the NURS complex required Red1, Iss10, or Rmn1 for stability (data not shown). Thus, Red1 and Iss10 are required for the NURS complex to concentrate in nuclear bodies. We note that *iss10^+^* deletion only modestly increases meiotic RNA levels (see below; Yamashita et al. 2013), suggesting that these bodies may improve the efficiency of meiotic RNA degradation, but that their assembly is not strictly required for this process.

To determine whether the Red1-containing nuclear bodies organize around the genomic loci of Red1 target genes, we marked the *mei4*^+^ locus with a *tet* operator (*tetO*) array and expressed a GFP-tagged Tet repressor (TetR-GFP). The array was inserted upstream of *mei4^+^*, but because the 3′ UTR of the *cdk9^+^* gene overlaps the 5′ UTR of *mei4^+^*, we duplicated the 567-bp region between the stop codon of *cdk9^+^* and start codon of *mei4^+^*so that it appears both upstream of and downstream from the array (Fig. 3C). The expression of *mei4^+^* and the levels of H3K9 methylation at this gene were unaffected by the presence of the *tetO* array and remained responsive to *red1*^+^ deletion (data not shown). We found that Red1-mCherry foci did not colocalize with the *mei4*^+^ locus (Fig. 3C). This result suggests that Red1 and its interacting partners assemble into nuclear bodies in regions that are distinct from sites of transcription. However, we cannot exclude the possibility of transient association of these bodies with meiotic gene loci.

### Mtl1, Iss10, and Red5 regulate meiotic mRNA degradation

**FIGURE 4.**
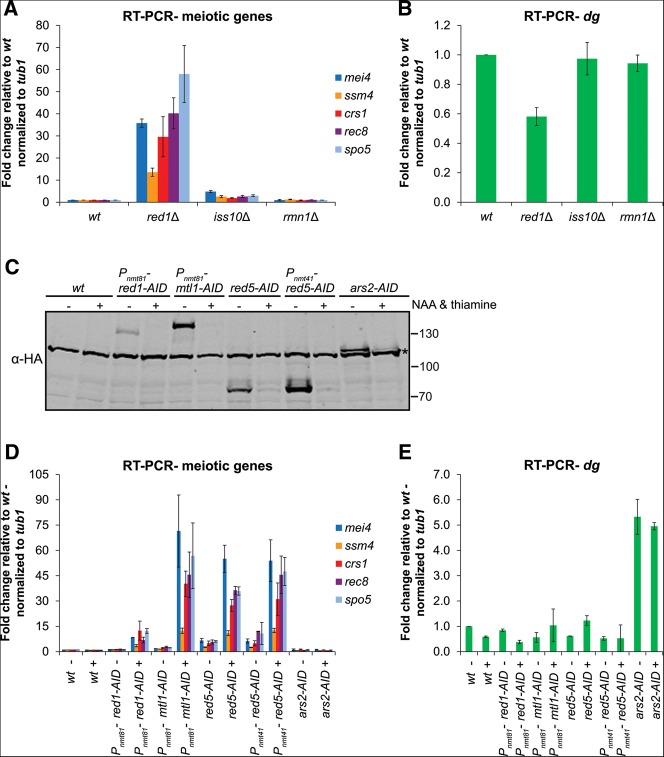
The roles of NURS complex subunits in RNA elimination. (*A*,*B*) Quantitative RT-PCR analysis of meiotic mRNA (*A*) and centromeric *dg* transcript (*B*) levels in wild-type cells and cells carrying deletions of the genes encoding the nonessential subunits of NURS. (*C*) Western blot showing the levels of auxin-inducible degron (AID)-tagged proteins, some driven by the thiamine-repressible *nmt* promoter, after the addition of DMSO (−) or thiamine and the synthetic auxin NAA (+). The asterisk indicates a protein that cross-reacts with the α-HA antibody. Molecular weight markers (kDa) are shown on the *right*. (*D*,*E*) Quantitative RT-PCR analysis of meiotic mRNA (*D*) and centromeric *dg* transcript (*E*) levels in wild-type cells and in cells expressing AID-tagged proteins after the addition of DMSO (−) or thiamine and NAA (+). Error bars represent standard deviation (SD). wt, wild-type.

We next investigated whether members of the NURS complex work with Red1 to degrade meiotic mRNAs. Deletion of *iss10*^+^ resulted in a modest increase in the levels of five different meiotic mRNAs, in good agreement with previous results (Fig. 4A; Yamashita et al. 2013). In contrast, deletion of *rmn1*^+^ had no impact on meiotic mRNA accumulation (Fig. 4A). The levels of an RNA Pol II transcript derived from the centromeric *dg* repeat were unaffected by *iss10*^+^ or *rmn1*^+^ deletion and only slightly reduced by *red1*^+^ deletion (Fig. 4B).

In order to examine the contributions of the essential proteins Mtl1, Red5, and Ars2 to meiotic RNA elimination, we utilized an auxin-inducible degron (AID) system. In plants, the hormone auxin binds to the TIR1 F-box protein component of a Skp1–Cullin–F-box (SCF) E3 ubiquitin ligase complex and promotes its association with a degron present in the AUX/IAA family of transcriptional repressors (Dharmasiri et al. 2005; Kepinski and Leyser 2005). The ubiquitinated repressor is then degraded by the proteasome, thus allowing the expression of auxin-responsive genes. Recently, Kanke and colleagues developed a strain of *S. pombe* expressing the TIR1 F-box proteins from *Arabidopsis thaliana* and *Oryza sativa* (rice) fused to the *S. pombe* Skp1, enabling the degradation of *S. pombe* proteins tagged with IAA degrons (Kanke et al. 2011). As in that report, we utilized the degron sequence from the *A. thaliana* IAA17 protein along with a 2×HA tag for Western blot detection (Kanke et al. 2011). We fused this degron tag to the C termini of the Mtl1, Red5, and Ars2 proteins, as well as the nonessential Red1 protein as a positive control. We also replaced the *red1*^+^, *mtl1*^+^, and *red5*^+^ gene promoters with a weakened version of the thiamine-repressible *nmt1* promoter (*nmt41* or *nmt81*) in order to combine protein depletion and transcriptional shut-off, a strategy successfully used previously (Basi et al. 1993; Kanke et al. 2011). However, we were unable to replace the promoter of *ars2*^+^ despite several attempts.

We added thiamine and the synthetic auxin 1-naphthaleneacetic acid (NAA) to exponentially growing cultures and observed a drastic reduction in AID-tagged protein expression (Fig. 4C). We then measured the levels of several meiotic mRNAs as well as a transcript derived from the centromeric *dg* repeats. The addition of thiamine and auxin had no effect on RNA levels in the TIR1-expressing parent strain (Fig. 4D,E). As expected, knockdown of Red1 significantly increased meiotic mRNA levels, although not to the same degree as *red1*^+^ deletion (Fig. 4A,D). Knocking down Mtl1 caused more dramatic increases in meiotic mRNA levels without affecting *dg* RNA levels (Fig. 4D,E). Similarly, depletion of Red5 resulted in meiotic mRNA accumulation, in agreement with a previous study of a temperature-sensitive *red5* mutant (Sugiyama et al. 2013), but did not alter *dg* RNA levels (Fig. 4D,E). Fusion of Mtl1 or Red5 to the degron tag, even without the addition of thiamine and NAA, also slightly increased meiotic mRNA levels, suggesting the tag alone interfered with their function (Fig. 4D). Finally, Ars2 knockdown did not affect meiotic gene expression, but fusion of this protein to the degron tag resulted in a modest (approximately fivefold) increase in the levels of *dg* RNA with or without the addition of thiamine and NAA (Fig. 4D,E). It is possible that residual levels of Ars2-AID are sufficient for degradation of meiotic mRNAs (Fig. 4C). Our results show that Red1, Mtl1, Iss10, and Red5 are required for meiotic mRNA degradation while Rmn1 and Ars2 are dispensable.

### NURS complex subunits link Red1 to Mmi1, the exosome, and splicing factors

**FIGURE 5.**
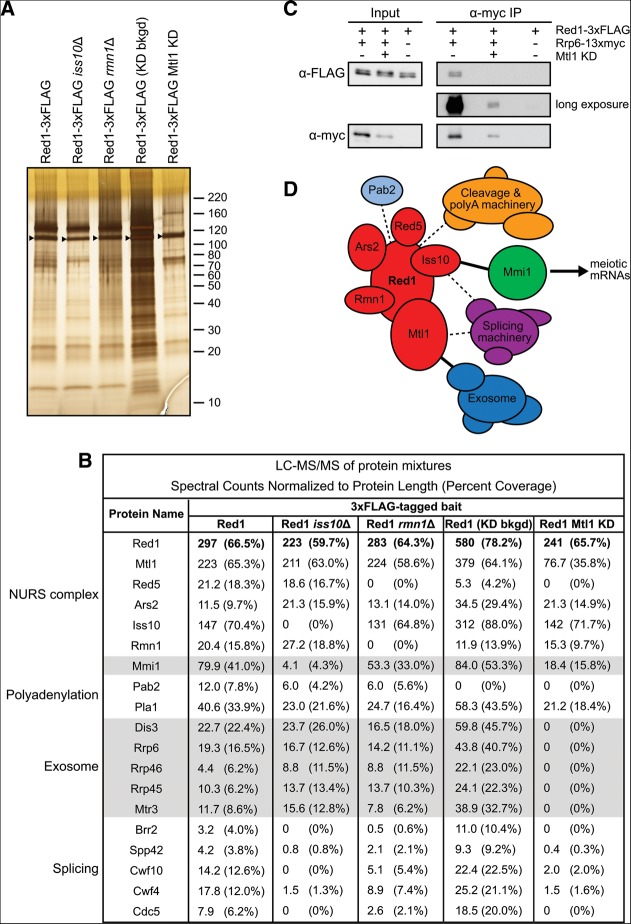
Requirements for integrity of the NURS complex and its association with other factors. (*A*) Silver-stained SDS polyacrylamide gels showing proteins recovered by purification of Red1-3×FLAG from wild-type cells, cells carrying deletions of *iss10*^+^ or *rmn1*^+^, or cells depleted of Mtl1. Arrowheads indicate the position of Red1-3×FLAG. Molecular weight markers (kDa) are shown on the *right*. (*B*) Results of mass spectrometry (LC-MS/MS) analysis of protein mixtures displayed as normalized spectral counts and percent coverage in parentheses. (*C*) Western blot showing co-immunoprecipitation of Red1-3×FLAG by Rrp6-13×myc in wild-type cells or cells depleted of Mtl1. (*D*) Proposed architecture of the NURS complex, highlighting the subunits that mediate its interactions with Mmi1, Pab2, the exosome, and the splicing machinery. Dashed lines represent interactions identified by purification and mass spectrometry and/or co-immunoprecipitation. Solid lines represent interactions bridged by the indicated protein based on the results of co-immunoprecipitation experiments in knockout or knockdown strains. KD, knockdown.

To dissect the mechanism of Iss10, Rmn1, and Mtl1 function, we purified Red1 in the absence of Iss10 or Rmn1 and after knocking down Mtl1 (Fig. 5A). While the levels of Red1 were unaffected (data not shown), we observed several changes in NURS complex interactions after the removal or depletion of these proteins. Deletion of *iss10*^+^ had no effect on the composition of the NURS complex but resulted in a near complete loss of Mmi1 association (Fig. 5B). This result confirms the previously reported dependence of Red1–Mmi1 co-immunoprecipitation on Iss10 (Yamashita et al. 2013) and further indicates that Iss10 acts as an adaptor that links the NURS complex to Mmi1. The deletion of *rmn1*^+^ disrupted the interaction between Red1 and Red5 without significantly affecting other Red1 interactions (Fig. 5B). The dependence of Red5 on Rmn1 for assembly into the NURS complex is puzzling, given that Rmn1 is not required for meiotic mRNA degradation while Red5 is (Fig. 4A,D). A likely explanation for this result is that the incorporation of Red5 into the NURS complex is weakened but not eliminated in *rmn1*Δ cells. Knockdown of Mtl1 also resulted in the loss of Red5 from the NURS complex, but Red5 was also poorly recovered in the control purification of Red1-3×FLAG from the parent strain (Fig. 5B). While knockdown of Mtl1 did not impact the assembly of the rest of the NURS complex, we observed a striking loss of multiple exosome subunits that were reproducibly identified in other Red1 purifications (Fig. 5B; Supplemental Table S3). Using co-immunoprecipitation and Western blotting, we confirmed the near complete loss of Red1–Rrp6 interaction after Mtl1 knockdown (Fig. 5C). This result, along with the finding that association of NURS with the exosome is not mediated by RNA (Fig. 2F,G; Sugiyama and Sugioka-Sugiyama 2011), suggests that Mtl1 links the NURS complex to the exosome and thus performs an analogous function to the Mtr4 subunit of the TRAMP complex (LaCava et al. 2005). Finally, knockdown of Mtl1 or deletion of *iss10*^+^ reduced Red1 association with splicing factors, suggesting that Mtl1 and Iss10 may stabilize interactions between the NURS complex and components of this pathway (Fig. 5B; Supplemental Table S3). Together these results provide insight into the architecture of the NURS complex and identify the Iss10 (via Mmi1) and Mtl1 subunits as adaptors that link the complex to meiotic RNAs and the exosome, respectively (Fig. 5D).

### Members of the NURS complex have distinct chromatin association patterns and effects on H3K9 methylation at meiotic genes

**FIGURE 6.**
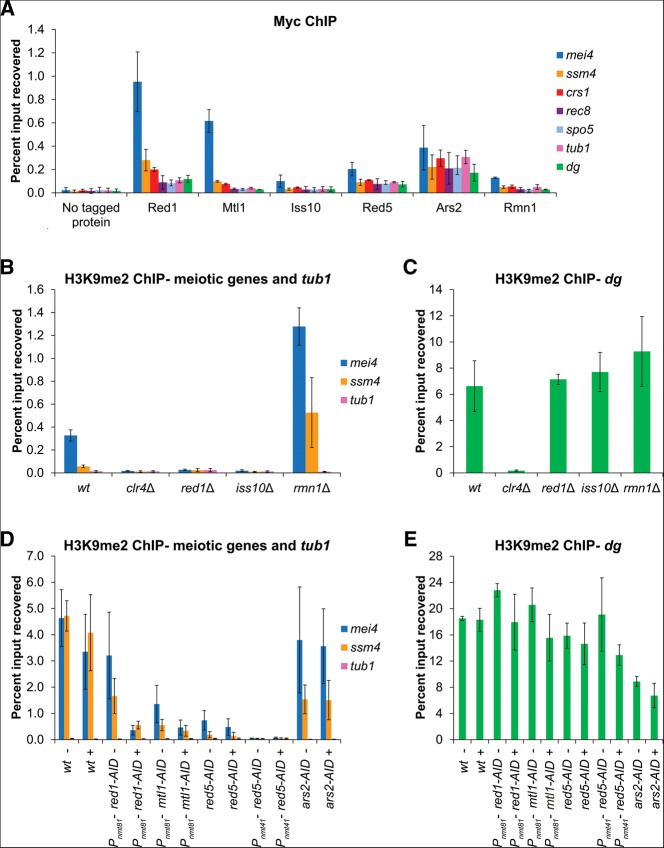
Association of the NURS complex with DNA and roles of NURS complex subunits in H3K9 methylation. (*A*) ChIP-qPCR analysis of 13×myc-tagged NURS complex subunit association with meiotic genes, the *tub1^+^* gene, and the centromeric *dg* repeat. (*B*,*C*) ChIP-qPCR analysis of H3K9me2 levels at meiotic genes and the *tub1^+^* gene (*B*) and the centromeric *dg* repeat (*C*) in the indicated wild-type and mutant cells. (*D*,*E*) ChIP-qPCR analysis of H3K9me2 levels at meiotic genes and the *tub1^+^* gene (*D*) and the centromeric *dg* repeat (*E*) in wild-type cells and cells expressing AID-tagged proteins, some driven by the thiamine-repressible *nmt* promoter, after the addition of DMSO (−) or thiamine and NAA (+). Error bars represent SD. wt, wild-type.

To begin to analyze the chromatin-related functions of the NURS complex, we tagged Red1, Mtl1, Iss10, Red5, Ars2, and Rmn1 with 13 myc epitopes and examined their association with several meiotic genes using chromatin immunoprecipitation (ChIP). In agreement with previous results (Zofall et al. 2012), we observed the greatest enrichment of Red1 at meiotic genes that possess detectable H3K9 methylation: *mei4*^+^, *ssm4*^+^, and *crs1*^+^ (Fig. 6A). This was also the case for Mtl1 (Fig. 6A). Iss10, Red5, and Rmn1 were enriched at *mei4*^+^ but did not exhibit a preference for other meiotic genes over *tub1*^+^ or centromeric *dg* repeats (Fig. 6A). Ars2 displayed a distinct pattern of chromatin localization, interacting similarly with all genes tested, including the euchromatic *tub1*^+^ locus and the heterochromatic *dg* repeats (Fig. 6A). This localization is likely mediated by an alternative pathway that does not recruit the remaining subunits of the NURS complex.

We next assessed the contribution of both the essential and nonessential subunits of the NURS complex to H3K9 methylation at meiotic genes. Deletion of *iss10*^+^ reduced H3K9 methylation at *mei4*^+^ and *ssm4*^+^ to near background levels but had no effect on H3K9 methylation at the centromeric *dg* repeats (Fig. 6B,C). This is the same phenotype as observed in *red1*Δ cells (Fig. 6B; Zofall et al. 2012). On the other hand, Rmn1 appears to function in restricting the amount of H3K9 methylation at meiotic genes since its absence caused elevated levels of H3K9 methylation at *mei4*^+^ and *ssm4*^+^ but not at the centromere (Fig. 6B,C).

The essential proteins Mtl1, Red5, and Ars2 were knocked down using the AID system followed by ChIP analysis. Importantly, the addition of auxin and thiamine had no effect on H3K9 methylation in the parent strain (Fig. 6D,E). Similar to *red1*^+^ deletion, knockdown of Red1 reduced meiotic gene H3K9 methylation (Fig. 6D). However, the levels of this modification at *ssm4*^+^ were lower than in the parent strain even before knockdown, suggesting the degron tag on Red1 interferes with its function in H3K9 methylation (Fig. 6D). Tagging of Mtl1 and Red5 also resulted in reduced H3K9 methylation levels at *mei4*^+^ and *ssm4*^+^, suggesting that they too contribute to H3K9 methylation (Fig. 6D). Knockdown of Mtl1 further reduced meiotic gene H3K9 methylation (Fig. 6D). Red5 depletion did not alter the very low levels of H3K9 methylation in the degron-tagged strain, but overexpression of the tagged protein using the *nmt41* promoter resulted in nearly undetectable levels of this modification (Fig. 6D). The tagging and knockdown of Ars2 did not affect H3K9 methylation at *mei4*^+^, but consistent with its requirement for full *dg* RNA silencing (Fig. 4E), did reproducibly decrease H3K9 methylation at the centromeric *dg* repeats and also at the *ssm4*^+^ gene (Fig. 6D,E). In addition, the fusion of Ars2 to the degron tag resulted in increased sensitivity to the microtubule-destabilizing drug thiabendazole (TBZ), a phenotype associated with the disruption of centromeric heterochromatin in *S. pombe* (Supplemental Fig. S2). However, *red1*Δ and *red5-AID* cells, as well as previously described temperature-sensitive *red5* mutant cells (Sugiyama et al. 2013), were also hypersensitive to TBZ, but exhibited wild-type centromeric silencing (Figs. 4B,E, 6C,E; Supplemental Fig. S2). TBZ sensitivity may therefore be an indirect consequence of defects in the silencing of meiotic genes or other unidentified targets. Our findings indicate that along with Red1, the Mtl1, Iss10, and Red5 subunits of the NURS complex are required to establish H3K9 methylation at meiotic genes while the Rmn1 subunit appears to oppose this modification. In addition, consistent with its more general chromatin association, Ars2 appears to have a broader function in promoting maximal H3K9 methylation at *ssm4*^+^ and at centromeric *dg* repeats.

### H3K9 methylation at meiotic genes does not affect expression

**FIGURE 7.**
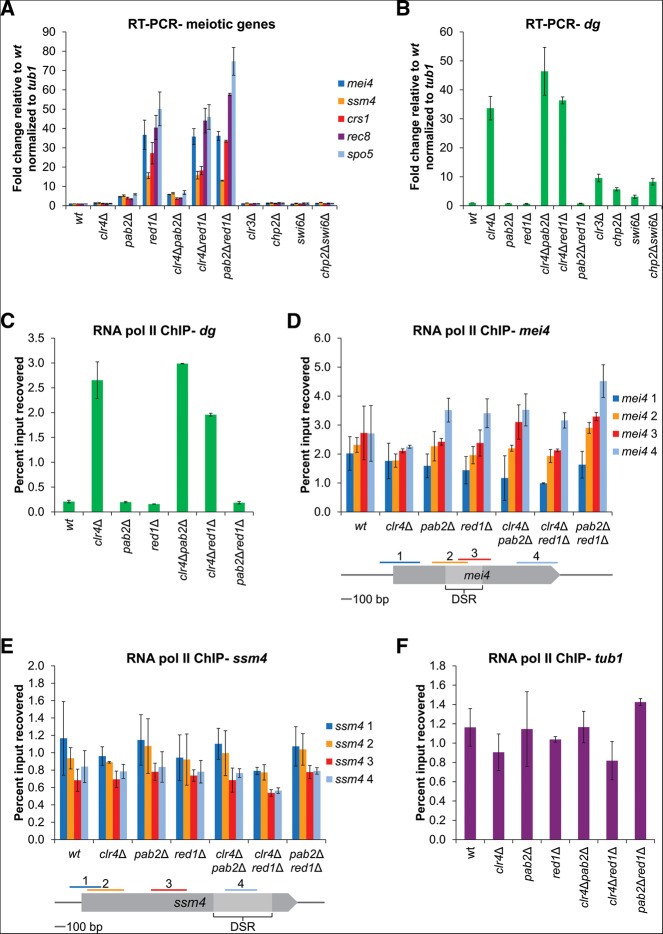
The impact of heterochromatin machinery on meiotic mRNA levels and RNA Pol II occupancy at meiotic genes. (*A*,*B*) Quantitative RT-PCR analysis of meiotic mRNA (*A*) and centromeric *dg* transcript (*B*) levels in wild-type cells and cells carrying the indicated deletions. (*C–F*) ChIP-qPCR analysis of RNA Pol II occupancy at the centromeric *dg* repeat (*C*), across the *mei4^+^* (*D*) and *ssm4^+^* (*E*) genes, and in the body of the *tub1^+^* gene (*F*). Error bars represent SD. wt, wild-type.

Finally, we assessed the impact of H3K9 methylation on meiotic gene expression. The levels of this repressive chromatin modification and of the HP1 protein Swi6 are lower at meiotic genes compared with regions of constitutive heterochromatin, the centromeres, telomeres, and mating type locus (Cam et al. 2005). Multiple groups have observed that the deletion of *clr4*^+^ does not affect meiotic mRNA levels, suggesting that the H3K9 methylation at these genes is either not sufficient for silencing or its effects are masked by the RNA elimination pathway (Zofall et al. 2012; Tashiro et al. 2013). In support of the second hypothesis, it was recently reported that *ssm4* RNA levels, as assayed by Northern blot, were higher in a *clr4*Δ*pab2*Δ double mutant compared with a *pab2*Δ single mutant (Zofall et al. 2012). We sought to extend this analysis by comparing the levels of several meiotic mRNAs, including those whose genes do and do not possess detectable H3K9 methylation, by quantitative real-time RT-PCR assays. We did not detect a significant additional increase in meiotic mRNA levels when the deletion of *clr4*^+^ was combined with deletions of either *pab2*^+^ or *red1*^+^ (Fig. 7A). We then investigated the effect of deleting heterochromatin factors that act downstream or together with H3K9 methylation and are required for transcriptional gene silencing. We did not observe a difference in the levels of any of the meiotic mRNAs in the absence of the Clr3 histone deacetylase or the HP1 proteins Swi6 and Chp2 (Fig. 7A). As a positive control, we also examined the levels of a centromeric *dg* transcript, which, as expected, were greatly increased in *clr4*Δ cells, as well as in *clr3*Δ, *chp2*Δ, and *swi6*Δ cells (Fig. 7B).

As an independent measure for the effect of H3K9 methylation on meiotic mRNA transcription, we also examined RNA Pol II occupancy across the *mei4*^+^ and *ssm4*^+^ genes in the absence of RNA elimination factors alone or in combination with deletion of *clr4*^+^. All strains lacking *clr4*^+^ exhibited a large increase in RNA Pol II occupancy at the centromeric *dg* repeats (Fig. 7C), as expected (Chen et al. 2008). However, no change in RNA Pol II occupancy was observed across meiotic genes or in the body of the *tub1*^+^ gene in any of the mutant strains (Fig. 7D–F). We conclude that the levels of H3K9 methylation and heterochromatin factors present at meiotic genes during vegetative growth are not sufficient to have a measurable impact on RNA Pol II accessibility or transcriptional output in wild-type cells, suggesting that the term “heterochromatin” may not accurately describe these regions of H3K9 methylation. However, in certain contexts, H3K9 methylation at meiotic genes may subtly influence expression. For example, the increases in *mei4* and *ssm4* RNA levels observed after overexpression of the anti-silencing factor *epe1*^+^ (approximately fourfold) or deletion of the RITS subunit *chp1*^+^ (1.5–2.5-fold) depend on the presence of *clr4*^+^ (Tashiro et al. 2013). Also, the H3K9 methylation-dependent recruitment of RITS subunits to meiotic genes is necessary for these proteins to weakly repress meiotic gene expression (Cam et al. 2005; Hiriart et al. 2012).

## DISCUSSION

We have identified a Red1-containing NURS complex whose members play distinct roles in the regulation of meiotic gene expression (Fig. 1). The Red1, Mtl1, Iss10, and Red5 subunits are required for the degradation of meiotic mRNAs and for H3K9 methylation at meiotic gene loci during vegetative growth (Figs. 4A,D, 6B,D). Ars2 and Rmn1 do not function in meiotic RNA elimination, but Rmn1 limits H3K9 methylation at meiotic genes and Ars2 contributes to centromeric silencing (Figs. 4A,D,E, 6B,D,E). Subunits of the NURS complex appear to link distinct RNA processing signals to exosome-mediated RNA degradation or histone H3K9 methylation. The conservation of NURS subunits and the recent identification of a human NURS-like complex suggest that this pathway plays a fundamental role in regulated nuclear RNA degradation (Lubas et al. 2011; Andersen et al. 2013).

An independent purification of Red1 was very recently reported (Lee et al. 2013). Although both studies identify a similar set of interacting proteins, key differences emerge when comparing the results of Lee and colleagues with those reported here. First, we identify Red5, which was recently found to interact with Pab2 and to function in RNA elimination (Sugiyama et al. 2013), as a subunit of the NURS complex, but Red5 was not identified in Red1 purifications performed by Lee et al. (2013). Second, we find that the NURS complex also includes Ars2, a protein reported, but not characterized, by the authors of that study due to its low abundance in their purifications of Red1, Mtl1, and Iss10 (Lee et al. 2013). Finally, Lee et al. (2013) proposed that Red1 and Mtl1 form a core that assembles at least three distinct complexes: one with Iss10 (renamed Pir1 in their study), a second with Pla1, and a third with Rmn1 and Pab2. In contrast, we find that Red1, Mtl1, Iss10, and Rmn1, along with Ars2 and Red5, form a single NURS complex. All members of this complex interact with Pab2 and Mmi1, and all except Red5 interact with Pla1 (Fig. 1C). These differences likely result from experimental variations that may disrupt the NURS holocomplex during cell extract preparation and sample processing. In addition to the NURS holocomplex, subcomplexes containing some, but not all, NURS subunits likely exist. In particular, our findings suggest the presence of a more stable Mtl1–Red1 core within the NURS complex, since purifications of Red1 and Mtl1 contain stoichiometric amounts of each other, as estimated by normalized spectral counts, while other subunits are co-purified in substoichiomertic amounts (Fig. 1C). We also observe that Mtl1 independently interacts with a small number of other proteins, not found in Red1 purifications, in good agreement with Lee and colleagues (Supplemental Table S1; Lee et al. 2013).

### Distinct pathways for targeting RNA to the exosome

**FIGURE 8.**
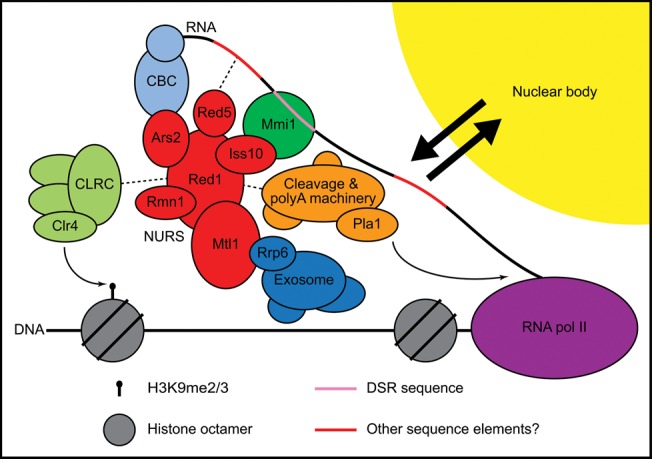
NURS complex interactions at meiotic genes. NURS, nuclear RNA silencing; CLRC, Clr4–Rik1–Cul4; CBC, cap-binding complex; DSR, determinant of selective removal.

Post-transcriptional RNA processing and degradation pathways are critical for the proper regulation of gene expression. Many of these pathways depend on exosome complexes, which exist in both the nucleus and cytoplasm (Chlebowski et al. 2013). In the nucleus, the Mtr4 helicase subunit of the TRAMP complex plays a central role in targeting specific RNAs for degradation by bridging RNA and the exosome (de la Cruz et al. 1998; LaCava et al. 2005). Our purification and analysis of the NURS complex reveal a second nuclear exosome targeting mechanism that involves an Mtr4-like protein, Mtl1 (Fig. 8). Mtl1 is specifically required for the association of the NURS complex with the exosome but not for the assembly of the NURS complex itself. In budding yeast, in addition to the nuclear TRAMP complex, a second complex containing the Mtr4-related helicase Ski2 targets RNAs to the exosome in the cytoplasm (Anderson and Parker 1998; Halbach et al. 2012). In human cells, a single Mtr4-like protein assembles into two distinct complexes, one of which is similar to the *S. cerevisiae* and *S. pombe* TRAMP complexes and a second, called the CBCN complex, which resembles the *S. pombe* NURS complex (Lubas et al. 2011; Andersen et al. 2013). The diversification of exosome targeting complexes containing Mtr4 and Mtr4-like proteins is evolutionarily conserved and is likely to be critical for the recognition and targeting of different RNA substrates to the exosome (Houseley et al. 2006; Chlebowski et al. 2013).

### NURS as an integrator of RNA processing signals

Our findings indicate that Mmi1 targets DSR-containing mRNAs to the exosome via association with the NURS complex (Fig. 7). In addition to such specific recruitment signals, exosome targeting is often linked to RNA-processing machineries, including factors involved in 5′ capping, splicing, transcription termination, 3′-end formation, and polyadenylation (Houseley et al. 2006). The degradation of meiotic mRNAs requires components of the 3′-end processing pathway such as the poly(A) polymerase Pla1 and the poly(A)-binding protein Pab2, both of which associate with the NURS complex (Fig. 1C; St-Andre et al. 2010; Yamanaka et al. 2010). Red1 and Mtl1 were also recently found to work with Pab2 to recruit the exosome subunit Rrp6 to specific small nucleolar (sno)RNAs and inefficiently spliced pre-mRNAs, including the ribosomal protein-encoding *rpl30-2* transcript. In the first case, the exosome promotes the trimming of the 3′ end to generate a mature snoRNA, while in the second, the pre-mRNA is degraded (Lemay et al. 2010; Lemieux et al. 2011; Lee et al. 2013). Our purification of Mtl1 also recovered the Nab2 poly(A)-binding protein (Supplemental Table S1), and a recent purification of Nab2 identified several NURS subunits (Grenier St-Sauveur et al. 2013). Nab2 appears to antagonize the action of Pab2, Rrp6, Red1, and Mtl1 in *rpl30-2* pre-mRNA degradation by competing with Pab2 for binding to the poly(A) tail (Grenier St-Sauveur et al. 2013; Lee et al. 2013). Additionally, the gene encoding another NURS subunit, *rmn1*^+^, exhibits a negative genetic interaction with *nab2*^+^ (Cho et al. 2012). Together, these data implicate the NURS complex in polyadenylation- and splicing-associated RNA processing and degradation pathways.

As noted above, the NURS complex is likely related to the human exosome-recruiting complex CBCN, which combines the RNA cap-binding and ARS2 complex (CBCA) and the nuclear exosome targeting (NEXT) complex (Fig. 8; Lubas et al. 2011; Andersen et al. 2013). CBCN and a separate TRAMP-like complex were identified by the purification of hMTR4, the human ortholog of *S. pombe* Mtl1 and Mtr4. The CBCN complex includes ARS2, the RNA cap-binding proteins CBP80 and CBP20, hMTR4, ZCCHC8, RBM7, and ZC3H18, and it associates with ZFC3H1 (Lubas et al. 2011; Andersen et al. 2013). *S. pombe* Ars2 is a subunit of the NURS complex, and this complex associates with the fission yeast cap-binding proteins Cbc1 and Cbc2 (Fig. 1C). Red1 is related to ZFC3H1, and in addition to possessing a zinc finger motif like Red1, ZFC3H1 has Pro/Ser-rich domains like Iss10. Thus, ZFC3H1 may combine the roles of Red1 and Iss10 in a single protein. Alternatively, ZCCHC8 and ZC3H18 also contain zinc finger motifs along with Pro-rich and Pro/Ser-rich domains, respectively, and could serve the functions of Red1 and/or Iss10. RBM7 is an RRM-containing protein that could fulfill the role of Rmn1, which also possesses this motif. Finally, the purification of hMTR4 also recovered several zinc finger- and YTH domain-containing proteins that may be orthologous to Red5 and/or Mmi1 (Lubas et al. 2011). In human cells, the CBCN complex recruits the exosome to degrade capped and polyadenylated promoter upstream transcripts (PROMPTs) (Lubas et al. 2011; Andersen et al. 2013; Hallais et al. 2013). CBCN also promotes the transcription termination and 3′-end processing of capped, but nonpolyadenylated, snRNAs, snoRNAs, and replication-dependent histone mRNAs (Andersen et al. 2013). The hMTR4 protein and the human ortholog of fission yeast Pab2, PABPN1, have also been implicated in the polyadenylation-dependent turnover of specific lncRNAs (Beaulieu et al. 2012). It will be of great interest to uncover the roles of the RNA cap and poly(A) tail in the activities of the NURS and CBCN complexes and to determine whether or not the CBCN complex functions in chromatin modification pathways.

### The NURS complex and histone H3K9 methylation

Connections between RNA processing/degradation and chromatin modification pathways continue to emerge. The TRAMP complex regulates centromeric silencing by processing centromeric transcripts and promoting their degradation by the exosome (Buhler et al. 2007; Reyes-Turcu et al. 2011). Splicing has also been implicated in heterochromatin formation in *S. pombe* (Bayne et al. 2008; Chinen et al. 2010; Lee et al. 2013). The NURS complex subunits Red1, Mtl1, Iss10, and Red5 are required for both meiotic mRNA degradation and H3K9 methylation at meiotic genes (Figs. 4A,D, 6B,D; Zofall et al. 2012; Lee et al. 2013; Sugiyama et al. 2013; Yamashita et al. 2013). Rmn1 appears to restrict H3K9 methylation at meiotic genes during vegetative growth but promotes the assembly of RNAi-dependent heterochromatin at specific loci in *rrp6*Δ cells in a pathway that also depends on Red1, Pab2, and Pla1 (Fig. 6B; Lee et al. 2013; Yamanaka et al. 2013). It is unclear how CLRC is recruited to target genes in these pathways, but the mechanism may involve direct interaction with Red1 (Zofall et al. 2012; Tashiro et al. 2013) or with other subunits of the NURS complex, several of which co-purify CLRC components (Fig. 1C).

In contrast to other members of the NURS complex, Ars2 plays a minor role in H3K9 methylation at meiotic genes, but has a greater impact on H3K9 methylation and silencing of centromeric *dg* repeats (Figs. 4E, 6D,E). In *S. pombe*, centromeric H3K9 methylation is largely RNAi-dependent, and the *Drosophila* and plant orthologs of Ars2, together with the cap-binding complex, have been shown to interact with Dicer proteins and Microprocessor components to regulate siRNA and miRNA biogenesis (Yang et al. 2006; Laubinger et al. 2008; Sabin et al. 2009; Raczynska et al. 2013). Our purification of Ars2 did not yield any components of the RNAi machinery but contained CLRC subunits (Fig. 1C). However, additional studies are required to determine whether Ars2 acts together with or independently of RNAi to promote efficient H3K9 methylation and silencing at pericentromeric DNA regions.

The H3K9 methylation at meiotic genes in vegetatively growing *S. pombe* cells has been likened to the facultative heterochromatin of multicellular organisms (Zofall et al. 2012; Tashiro et al. 2013). However, the levels of this modification at meiotic genes are very low compared with those found at regions of constitutive heterochromatin such as the centromere (Fig. 6B–D; Cam et al. 2005; Tashiro et al. 2013). We found that meiotic mRNA levels were unchanged in the absence of heterochromatin factors (Fig. 7A) and that deletion of *clr4^+^*, *pab2^+^*, and *red1^+^*, alone or in combination, did not affect RNA Pol II occupancy at meiotic genes (Fig. 7D,E). Furthermore, the NURS complex and exosome both localize to nuclear bodies (Fig. 3; Yamanaka et al. 2010; Sugiyama and Sugioka-Sugiyama 2011) and can be cross-linked to the *mei4^+^*and *ssm4^+^* genes in ChIP experiments (Fig. 6A; Zofall et al. 2012; Lee et al. 2013), but the nuclear bodies do not colocalize with the *mei4^+^* locus (Fig. 3C). Together, these findings suggest that silencing of meiotic genes occurs primarily at steps that follow the initiation of transcription. We propose that the NURS complex associates with meiotic RNAs co-transcriptionally and promotes their transfer to the nuclear bodies where they are degraded by the exosome (Fig. 8). It remains unclear whether *S. pombe* utilizes the NURS complex to assemble functional heterochromatin at meiotic genes (perhaps in response to undefined signals), or whether the low levels of H3K9 methylation regulate the timing of meiosis by delaying the full-scale induction of meiotic genes. Alternatively, H3K9 methylation at meiotic genes may result from vestigial association of the CLRC and NURS complexes, which may have been critical at some point during evolution to silence these genes or other chromosome regions such as the pericentromeric repeats.

## MATERIALS AND METHODS

### Strains and plasmids

All strains were constructed using a PCR-based gene targeting approach (Bahler et al. 1998) and are listed in Supplemental Table S4. To make a strain expressing GFP-3×FLAG as a negative control for FLAG purification/mass spectrometry experiments, a pFA6a-hphMX6-P_*nmt41*_-GFP-3×FLAG plasmid was generated by first replacing the kanMX6 marker of pFA6a-kanMX6-P_*nmt1*_-GFP with hphMX6 then performing DpnI mutagenesis to convert the *nmt1* to the *nmt41* promoter (Li and Wilkinson 1997). DpnI mutagenesis was then used to replace the stop codon of GFP with an in-frame BamHI site to allow insertion of the 3×FLAG sequence PCR-amplified from pFA6a-3×FLAG-kanMX6 with flanking BamHI and XhoI sites. The hphMX6-P_*nmt41*_-GFP-3×FLAG cassette was inserted into the *S. pombe* genome 1 kB upstream of the *ura4*^+^ start codon. The pFA6a-mCherry-kanMX6 plasmid was made by amplifying the mCherry coding sequence with flanking PacI and AscI sites and inserting the fragment into pFA6a-kanMX6. To generate pFA6a-2×HA-AID(IAA17)-kanMX6, the degron coding sequence was PCR-amplified with flanking BamHI and AscI sites and inserted into pFA6a-3×HA-kanMX6. The pFA6a-hphMX6-P_CMV_-TetR-GFP plasmid was created by cutting pFA6a-hphMX6-P_*nmt1*_-GFP with BglII and PacI to remove the *nmt1* promoter and inserting a PCR fragment containing the CMV promoter, *tet* repressor and λcI linker with flanking BamHI and PacI sites amplified from the genomic DNA of strain NBY3771 (Zilio et al. 2012). The hphMX6-P_CMV_-TetR-GFP cassette was inserted into the *S. pombe* genome at the BamHI site downstream from the *ura4-D18* locus (813 bp after the stop codon in *ura4*^+^ strains).

To integrate the *tetO* repeat array upstream of *mei4*^+^ without disrupting *mei4*^+^ expression, a pFA6a-*ura4*^+^-P_*mei4*_ plasmid was constructed in two steps. First, a 567-bp fragment containing the region between the *cdk9*^+^ stop codon and *mei4*^+^ start codon was PCR-amplified from genomic DNA with flanking PciI and NcoI sites and inserted into the PciI site of pFA6a-kanMX6. The kanMX6 cassette was then replaced with the 1.8-kB *ura4*^+^ fragment from KS-*ura4*^+^ (Bahler et al. 1998). A PCR product including the target regions (Rohner et al. 2008), *ura4*^+^ cassette, and *mei4*^+^ promoter flanked by 80 bp homologous to the genomic sequence immediately upstream of the *mei4*^+^ coding region was used to transform a *ura4-D18* strain expressing GFP-tagged Tet repressor (TetR), and transformants were selected on medium lacking uracil. The pSR14 plasmid (Rohner et al. 2008), which contains ∼324 copies of the *tet* operator (*tetO*), was modified to replace the *LEU2* marker with the natMX6 cassette (Hentges et al. 2005). A linearized pSR14-natMX6 plasmid with left and right target regions homologous to those flanking the integrated *ura4*^+^ marker was used to transform the strain described above, and transformants were selected with clonNAT (Werner BioAgents) and then screened for uracil auxotrophy.

### Protein purification and mass spectrometry

Cell extracts for large-scale affinity purifications were prepared based on the method described (Oeffinger et al. 2007). Three liters of cells grown to a density of 6 × 10^7^ cells/mL at 32°C in yeast extract medium with supplements (YES) were harvested by centrifugation. Cell pellets were resuspended in a volume of lysis buffer (20 mM HEPES pH 7.5, 100 mM NaCl, 5 mM MgCl_2_, 1 mM EDTA, 10% glycerol, 1 mM PMSF) equal to one-fifth of the pellet volume then added dropwise to liquid nitrogen. Cells were lysed by three cycles of cryo-milling at 30 Hz for 3 min each in a Retsch MM301 mixer mill. Yeast powder was resuspended in a volume of IP buffer (lysis buffer plus 0.25% Triton X-100, 0.5 mM DTT, and cOmplete protease inhibitor cocktail [Roche]) equal to 1.2× the pellet volume and centrifuged at 16,000*g* for 5 min followed by filtration as described (Oeffinger et al. 2007) or by two additional rounds of centrifugation at 16,000*g* for 15 min each. For each sample, 275 μL of prewashed Protein G Dynabeads (Life Technologies) were incubated with 38 μg of α-FLAG (M2, Sigma) or α-myc (9E10, Covance) antibody overnight at 4°C. Antibodies were cross-linked to the beads using 15 mM dimethyl pimelimidate (Pierce) in 0.2 M sodium borate, pH 9. Crosslinking was allowed to proceed for 30 min at room temperature and was quenched by the addition of 0.2 M ethanolamine, pH 8. Beads were washed twice with 10 volumes of IP buffer and resuspended in 1 volume of IP buffer before adding to cell extracts and incubating at 4°C for 3 h. Beads were washed with 1 mL ice cold IP buffer three times for 1 min each followed by one 5-min wash. Proteins were eluted in 687.5 μL of 500 mM ammonium hydroxide at 37°C for 20 min and samples were dried overnight using a SpeedVac concentrator (Savant). Immunoprecipitated proteins were resuspended in 75 μL of buffer containing 100 mM Tris, pH 8.2, and 2 M urea. DTT was added to a final concentration of 5 mM, and samples were incubated at 56°C for 30 min. After cooling to room temperature, iodoacetamide was added to a final concentration of 15 mM, and samples were incubated in the dark for 45 min. Unreacted iodoacetamide was quenched with addition of DTT to a final concentration of 10 mM. Samples were diluted with 100 mM Tris, pH 8.2 to bring the urea concentration to 1 M, and sequencing-grade trypsin (Promega) was added at a concentration of 15 ng/μL followed by incubation at 37°C overnight. Digested samples were then loaded onto stage tips and washed as described previously (Rappsilber et al. 2007). Peptides were eluted with 50% acetonitrile, 0.5% acetic acid, dried using a SpeedVac concentrator (Savant), and resuspended in 1% formic acid. Resuspended samples were analyzed by liquid chromatography (LC)-MS/MS by infusing from a C18 column (18 cm by 125 μm [inner diameter]) into either an LTQ Orbitrap XL (Thermo) or Q Exactive (Thermo) hybrid mass spectrometer. Spectra were acquired in a data-dependent fashion. The resulting MS/MS spectra were recorded for each run and then searched against a target-decoy database of *S. pombe* tryptic peptides using SEQUEST (Elias and Gygi 2007). The final peptide list was filtered to a false positive rate of not greater than 2% at both the peptide and protein level.

### Co-immunoprecipitation and Western blotting

Cells were grown to a density of 4 × 10^7^ cells/mL at 32°C in YES or in yeast extract medium containing 225 mg/L adenine (YEA) and harvested by centrifugation. Pellets from ∼1.25 × 10^10^ of cells were resuspended in 600 μL of lysis buffer and lysed by six rounds of agitation with glass beads at 4500 rpm for 30 sec each in a MagNA Lyser instrument (Roche). Cell lysates were subjected to two rounds of centrifugation at 13,000*g* for 5 and 15 min. Approximately 20 mg of total protein was combined with 3 μg α-myc antibody (9E10, Covance) prebound to 20 μL Protein G Dynabeads (Life Technologies) in a total volume of 700 μL and incubated at 4°C for 2–3 h. Beads were washed three times in 1 mL lysis buffer for 5–10 min each, and bound proteins were eluted by incubation at 65°C for 8 min in loading buffer (50 mM Tris-HCl pH 6.8, 2% SDS, 10% glycerol, 100 mM DTT, and bromophenol blue). RNase A was added to lysis buffer at a final concentration of 100 μg/mL and was present during both the binding and washing steps. For protein knockdown experiments, denatured whole cell extracts were prepared as described previously (Knop et al. 1999). Proteins were separated by SDS-PAGE and transferred to a nitrocellulose membrane (Whatman) followed by incubation with horseradish peroxidase (HRP)-conjugated α-FLAG (A8592, Sigma), α-myc (R951-25, Life Technologies), or α-HA (H6533, Sigma) primary antibody. In Figures 2 and 5C, direct detection of the α-FLAG-HRP or α-myc-HRP primary antibody was performed using SuperSignal West Chemiluminescent Substrate (Pierce) and a Fujifilm LAS-3000 imager. In Figure 4C and Supplemental Figure 1, blots were incubated with α-mouse secondary antibody conjugated to IRDye800 (Rockland) and tagged proteins were detected using an Odyssey scanner (LI-COR).

### Fluorescence microscopy

Cells expressing GFP- and mCherry-tagged proteins were grown to logarithmic phase at 25°C in Edinburgh minimal medium supplemented with 100 mg/L adenine, leucine, histidine, lysine, and uracil (EMMC). Cells were pipetted onto slides and covered with a coverslip immediately prior to imaging using a Nikon Ti-E inverted epi-fluorescence microscope equipped with a Plan Apo 100× 1.4 NA objective lens. A Prior LumenPro light source and 490/20 and 572/35 excitation filters (Chroma) were used to excite GFP and mCherry fluorescence, respectively. Emission was collected using a 535/30 filter for GFP and a 630/60 filter for mCherry (Chroma). Images were acquired using a Hamamatsu ORCA-R2 cooled CCD camera and the Nikon Ti focus motor, controlled with MetaMorph v7.7 software (Molecular Devices). Single plane images are displayed, with brightness and contrast adjusted using MetaMorph.

### Protein knockdown using the auxin-inducible degron (AID) system

Strains expressing target proteins tagged with 2×HA-AID(IAA17) and fusions of *S. pombe* Skp1 to the *A. thaliana* and *O. sativa* TIR1 F-box proteins (Kanke et al. 2011) were grown to saturation overnight at 32°C in EMMC. Cultures were diluted to ∼9 × 10^6^ cells/mL and shifted to 25°C for 4 h before the addition of thiamine and 1-naphthaleneacetic acid (NAA, Sigma) to final concentrations of 15 μM and 500 μM, respectively. Control cultures were treated with the same volume of DMSO (NAA vehicle). Cells were returned to 25°C for 6 h before processing for ChIP, Western blotting, co-immunoprecipitation, and RNA analysis and for 14 h for purification/mass spectrometry.

### RNA analysis

Total RNA was extracted from exponentially growing *S. pombe* cells using the hot phenol method (Leeds et al. 1991). Twenty-five micrograms of RNA was treated with DNase I (Roche) and purified using the RNeasy mini kit (Qiagen). Reverse transcription was performed using Superscript III reverse transcriptase (Life Technologies) and gene-specific primers. Samples were then analyzed by quantitative PCR using the Applied Biosystems 7900HT Fast Real-Time PCR System. See Supplemental Table S5 for oligonucleotide sequences.

### Chromatin immunoprecipitation (ChIP)

Cells grown to a density of 2.5 × 10^7^ cells/mL at 32°C in YEA were cross-linked with 1% formaldehyde for 30 min at room temperature followed by quenching with glycine at a final concentration of 125 mM and washing as described (Huang and Moazed 2003). Pellets from ∼6.5 × 10^8^ of cells were resuspended in 400 μL of lysis buffer (50 mM HEPES-KOH pH 7.5, 140 mM NaCl, 1 mM EDTA pH 8.0, 1% Triton X-100, 0.1% sodium deoxycholate, 1 mM PMSF, and cOmplete protease inhibitor cocktail [Roche]) and lysed by three rounds of agitation with glass beads at 4500 rpm for 45 sec each in a MagNA Lyser instrument (Roche). Cell lysates were sonicated and clarified by centrifugation as described (Huang and Moazed 2003). After removal of an input sample, a volume of 400 μL of cell lysate was combined with 2.5 μg of α-H3K9me2 (ab1220, Abcam), α-RNA Pol II antibody (8WG16, Covance), or α-myc (9E10, Covance) antibody prebound to 30 μL of Protein A or G Dynabeads (Life Technologies) in a total volume of 500 μL. Samples were rotated at 4°C for 2–3 h. Beads were washed twice in 1 mL of lysis buffer, once in lysis buffer containing 500 mM NaCl, and once in TE (10 mM Tris-HCl pH 8.0 and 1 mM EDTA pH 8.0) at room temperature for 5 min each. Complexes were eluted as described (Huang and Moazed 2003). Input and IP samples were incubated at 65°C overnight, combined with 250 μL of TE and 50 μg RNase A, and incubated at 37°C for 30 min. Then 60 μg of glycogen and 100 μg of Proteinase K (Roche) were added, and samples were incubated at 37°C for 2 h. DNA was purified and analyzed by quantitative PCR using the Applied Biosystems 7900HT Fast Real-Time PCR System. See Supplemental Table S5 for oligonucleotide sequences.

### Thiabendazole (TBZ) assay

Cells grown overnight at 30°C were resuspended in water at a density of 1 × 10^8^ cells/mL. Three microliters of serial 10-fold dilutions were spotted onto nonselective YES medium and YES supplemented with 17 μg/mL TBZ. Plates were incubated at 32°C for 3–4 d, then photographed.

## SUPPLEMENTAL MATERIAL

Supplemental material is available for this article.
